# Optical Coupling in Atomic Waveguide for Vertically Integrated Photonics

**DOI:** 10.34133/research.0329

**Published:** 2024-03-11

**Authors:** Yue Wang, Junzhuan Wang, Ruijuan Tian, Jiapeng Zheng, Lei Shao, Bo Liu, Fengqiu Wang, Xuetao Gan, Yi Shi, Xiaomu Wang

**Affiliations:** ^1^ School of Electronic Science and Engineering, Nanjing University, Nanjing 210093, China.; ^2^ Key Laboratory of Light Field Manipulation and Information Acquisition, Ministry of Industry and Information Technology, and Shaanxi Key Laboratory of Optical Information Technology, School of Physical Science and Technology, Northwestern Polytechnical University, Xi’an 710129, China.; ^3^ Department of Physics, The Chinese University of Hong Kong, Hong Kong SAR, China.; ^4^ State Key Laboratory of Optoelectronic Materials and Technologies, Guangdong Province Key Laboratory of Display Material and Technology, School of Electronics and Information Technology, Sun Yat-sen University, Guangzhou 510275, China.; ^5^ Institute of Optics and Electronics, Nanjing University of Information Science and Technology, Nanjing 210044, China.

## Abstract

Integrated 2-dimensional (2D) photonic devices such as monolayer waveguide has generated exceptional interest because of their ultimate thinness. In particular, they potentially permit stereo photonic architecture through bond-free van der Waals integration. However, little is known about the coupling and controlling of the single-atom guided wave to its photonic environment, which governs the design and application of integrated system. Here, we report the optical coupling of atomically guided waves to other photonic modes. We directly probe the mode beating between evanescent waves in a monolayer 2D waveguide and a silicon photonic waveguide, which constitutes a vertically integrated interferometer. The mode-coupling measures the dispersion relation of the guided wave inside the atomic waveguide and unveils it strongly modifies matter’s electronic states, manifesting by the formation of a propagating polariton. We also demonstrated light modulating and spectral detecting in this compact nonplanar interferometer. These findings provide a generalizable and versatile platform toward monolithic 3-dimensional integrated photonics.

## Introduction

Recently, 2-dimensional (2D) materials–integrated photonics emerges as a promising technology for inter- and intrachip optical communications, as it clearly demonstrates the potential in developing optoelectronic devices with low cost, compact footprint, and high bandwidth [1–5]. The rich optical properties of 2D materials along with their van der Waals (vdW) heterostructure extends the operation wavelength and promotes the performance of integrated photonics [6–14]. These tunable-bandgap and ultrafast-response layered materials have been used for light generation, detection, and modulation, such as light emitters, lasers, modulators, and on-chip broadband photodetectors. Their layered properties give rise to a variety of novel heterostructures in the form of vdW stacks, with lateral, juxtaposed, or vertical 3-dimensional (3D) architectures. They exhibit unique light–matter interactions and polarization manipulation capabilities with promising applications in optoelectronics and twistronics [15]. Remarkably, 2D material films are also able to act as waveguides (planar or with delta film), even in an ultimately single atomic thickness, taking advantage of the high refractive index and low-loss material properties [16–18].

3D photonic integration is long expected and introduces a new degree of freedom in the design of photonic integrated circuits with compact size. Despite the traditional optical component, the 2D material film with their thinnest geometry size and versatile vdW integration ability provide a new opportunity toward monolithic 3D integrated photonic platforms. However, to achieve such a goal (or even simply to apply it to any photonic platform), it is necessary to elucidate the coupling between single-atom-thick waveguide/component and other photonic modes when assembling them into the integrated system.

Different from previous work, which has only focused on isolated monolayer waveguides, in this article, we vertically assemble the atomically thin waveguide with another integrated photonic component and study the optical coupling of the single-atom guided wave to the photonic environment, thus building a vertically integrated interferometer. We have studied the crucial role played by evanescent surface coupling. Using this 3D integrated photonic platform, we elucidated the mode coupling for the surface wave inside the atomic waveguide. In addition, we also explored how to control the mode coupling in the interferometer (by both field effect and finely tuned structure) and obtained a compact interferometric modulator and a spectrally resolved photodetector. These results not only provide a deep insight to understand single-atom-thick optical components but also inspire advanced integrated photonic platforms.

## Results and Discussion

**Fig. 1.**
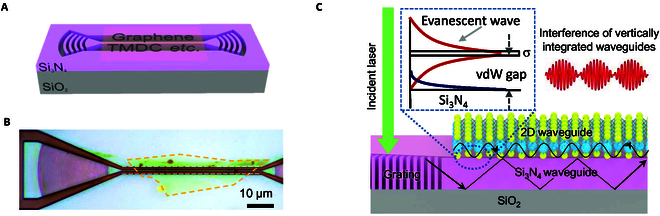
Schematics of the vertically integrated interferometer. (A) Schematic of the interferometer. It comprises a noninsulating monolayer 2D waveguide (such as graphene, MoS_2_, WSe_2_, etc.) and a silicon nitride waveguide. (B) Optical micrograph of a typical device. Dashed lines indicate WSe_2_ flake boundaries. (C) Working mechanism of the interferometer. The guided wave inside the top atomic waveguide evanescently interferes with the surface wave of bottom silicon nitride waveguide, generating a mode beating. Inset: Schematic of a transverse profile of light fields at the interface between 2D and silicon nitride waveguides. *σ* is the optical conductivity of 2D materials.

Figure 1A schematically shows the prototype vertical integrated interferometer device. It is composed of 2 vertically stacked waveguides, namely, an atomic 2D film waveguide above a silicon nitride ridge waveguide (see Methods for detailed device fabrication). We tested different 2D materials including graphene, WSe_2_, MoS_2_ monolayers, and

 thin-hexagonal-boron-nitride-encapsulated transition metal dichalcogenides (TMDCs). All of the results are qualitatively consistent. For simplicity, we mainly used WSe_2_ (as shown in Fig. 1B) as an example below otherwise mentioned.

For the operation, the incident laser is firstly fed into the lower waveguide through a grating. At the edge of 2D materials, the scattered beam excited a highly confined wave inside the upper atomic waveguide. The wave can be understood by considering a propagating transverse electric (TE) mode as illustrated in Fig. 1C With the 2D boundary condition (transverse magnetic [TM] mode yield qualitatively similar results), the dispersion of this surface wave can be expressed as:

$$\frac{\varepsilon_{1}}{\sqrt{k^{2}-\varepsilon_{1}\frac{\omega^{2}}{c^{2}}}}+\frac{\varepsilon_{2}}{\sqrt{k^{2}-\varepsilon_{2}\frac{\omega^{2}}{c^{2}}}}=-\frac{\mathrm{i\sigma}}{{\omega \varepsilon }_{0}}$$ (1)

where *k* is the wavevector, *ω* is the wave frequency, *ε*_i_ (i = 1,2) is the dielectric constant of top and bottom materials, and *σ* is the optical conductivity of 2D materials [19]. It is worth mentioning that all noninsulating 2D materials (with positive imaginary part of *σ*) universally support this kind of surface wave (see Supplementary Text 1 for MoS_2_ [Fig. S1] and graphene [Fig. S2] cases, respectively). Because the vdW gap that separates the 2 waveguides is very small (~0.5 nm for pristine 2D materials and a few nanometers for hexagonal-boron-nitride-sandwiched samples), the waves inside from the 2 waveguides are expected to strongly interact with each other.

**Fig. 2.**
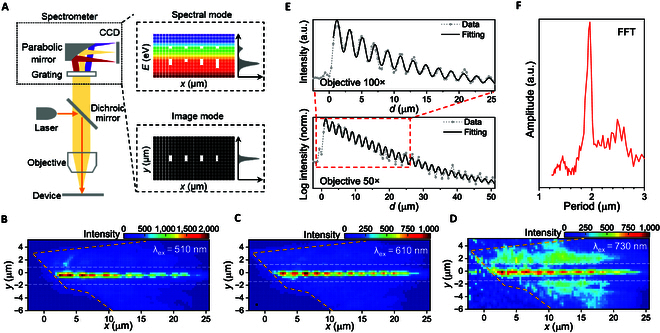
Real-space PL imaging of atomic waveguide interference. (A) Illustration of the experimental setup where the imaging mode of a charge-coupled device (CCD) camera is switched by angle of a spectrometer grating. (B to D) Selected real-space PL imaging data of the device shown in Fig. 1B, excited by various laser wavelengths. The yellow and white dashed lines mark the WSe_2_ flake boundaries and waveguide edges, respectively. The periodic oscillation reflects the interference between silicon nitride and atomic waveguides. (E) PL line profile along the middle of waveguide in (C) with different lens magnification. Here, *d* is the distance along the waveguide from the start of WSe_2_ flake edge. The open circles are measured results, the black lines are oscillation fittings. (F) Fast Fourier transform of the real-space PL profile in (E), showing the oscillation period.

We then used a real-space interferometric imaging technique to examine the optical coupling between the 2 waveguides as shown in Fig. 2A The key idea is that a plethora of luminescent sources (e.g., Raman and excitonic photoluminescence [PL], etc.) in 2D materials are able to play as probes to detect the spatial light field distribution. We used PL emission of WSe_2_ A exciton (~1.65 eV) in this case. Specifically, emitted PL signals were focused onto a grating spectrometer equipped with a charge-coupled device camera by an objective lens. The spectrometer was operated at either spectroscopic mode (to analysis the PL spectra along the waveguide) or imaging mode (to map the PL emission in real space) depending on the grating angle.

Figure 2B to D shows a typical set of PL images excited by different laser wavelengths. Generally, the PL signals exponentially decay along the propagation direction, reflecting a regular light absorption with constant absorption coefficient. In addition, we also observed periodically oscillated PL signals superimposed on the exponentially decayed base line. Notably, the oscillation presents a typical evanescent feature: it rapidly decreases with TMDC thickness and thus can be only observed in very thin samples (<5 layers; Supplementary Text 2). In a monolayer case, the oscillation stably propagates along the waveguide on a long propagation length (more than several tens of micrometers) under ambient conditions, as shown in Fig. 2E. In summary, we found the total PL intensity *I* as a function of propagation length *x* well fits to *I* ∝  sin (*βx*) × *e*^−*αx*^ where *α* = 0.07 μm^-1^ is the in-plane absorption coefficient (It should be noted that this in-plane loss originates from A exciton because we use it as a probe for the characterization. However, the 2D waveguide can be intrinsically lossless; see graphene in Supplementary Text 1 for instance.) and *β* is a wavelength-dependent oscillation period.

We attribute the periodic oscillation to a mode beating between 2D and silicon nitride waveguide modes, which directly reflects their interference (as illustrated in Fig. 1C). Note that the 2 modes from upper and lower waveguides are with different *E* − *k* (energy–wavevector) dispersions. Owing to the existence of a wavevector mismatch Δ*k* = *k*_*WSe*2_ − *k_SiN_* at a fixed energy between the 2 photonic modes, a mode interference is created. In other words, the amplitude of the sum signal is spatially modulated, featured by a periodic envelope, and embodied in the far-field emission. Accordingly, we unveiled that the surface waves of the 2 waveguides evanescently interfere with each other, resulting in a vertically integrated interferometer. Note that the TM mode gives a similar interference pattern as that of the TE mode (see Supplementary Text 3 and Fig. S4). It is quite natural because the TM interference also originates from mode beating (i.e., between 2D waveguide and silicon nitride TM mode). Only the periods differ slightly, with 1.99 μm for TE and 1.63 μm for TM. This difference results from the slightly different effective index for waveguide TE and TM modes.

**Fig. 3.**
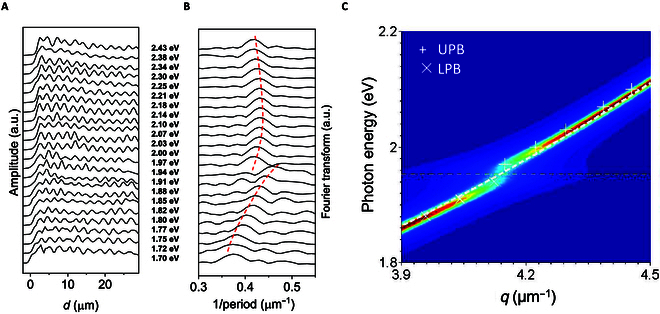
Propagating exciton–polariton inside the atomic waveguide. (A and B) Real-space PL profiles (A) and the corresponding Fourier transform profiles (B) of a typical TMDC vertically integrated interferometer taken at various excitation energies (1.70 to 2.43 eV). All the profiles are displaced vertically for clarity. The dashed lines in (B) guide the main peaks. Its anti-crossover behavior indicates the formation of a polariton. (C) Dispersion map of exciton–polariton calculated by using the mode coupling theory. The white and yellow dash lines presents the modeled dispersion of atomic waveguide TE_00_ mode and A exciton of WSe_2_, respectively. The experimentally measured wavevector values of mode beating in (B) are transferred to 2 branches of exciton polariton (LPB, lower polariton branch; UPB, upper polariton branch) and superimposed onto the map showing good agreement.

We then utilize the interferometer to explore the optical properties of the wave inside the atomic waveguide. Remarkably, the oscillation period of the interferometric envelope is much larger than the wavelength of original PL due to that it corresponds to a small Δ*k*. The detectable interference pattern thus permits to characterize detailed *E* − *k* dispersion of the atomically guided wave. For this purpose, we changed the excitation laser wavelength and measured the dispersion of the oscillation (see Methods). Figure 3A plots the PL intensity (of WSe_2_ A exciton) profiles along the interferometer. The excitation photon energies ranges from 1.70 to 2.43 eV. The corresponding Fourier transform curves are shown in Fig. 3B, where we can extract Δ*k* for each photon energy. Interestingly, the dispersion presents an obvious anti-crossover (split at WSe_2_ B exciton of ~2eV) behavior, suggesting the formation of an exciton–polariton as a mixture of semilight and semimatter states. It results from strong coupling between the photonic mode and exciton quasi-particle inside the 2D materials [16,20,21].

As aforementioned, the optical conductivity *σ* determines the photonic mode inside the atomic waveguide. We thereby focus on the *σ* arising from the exciton. From the perspective of quantum mechanics, elementary excitations give rise to imaginary parts (*ε*˝) of dielectric function [22]. For the WSe_2_ case, *ε*˝ fits well to a Lorentz oscillator as a result of the excitonic effect, namely

$$\varepsilon^{\prime \prime }=M{\left|U\right|}^{2}\frac{\Gamma /2}{{\left(E_{\mathrm{ex}}-\hbar \omega \right)}^{2}+{\left(\frac{\Gamma }{2}\right)}^{2}},$$ (2)

where *ω* is the frequency, *ħ* is the Planck’s constant, *M* is an optical transition matrix element characterizing the transition rate from the initial to final states, *U*^2^ represents the effect of excitons on the oscillator strength of the interband transition, *E_ex_* is the exciton energy involved, and *Γ* is a damping constant determining the bandwidth of the interband transition [23]. Under this circumstance, *σ* can be deduced from $\varepsilon =1+\frac{\mathrm{i\sigma}}{{\omega \varepsilon }_{0}}$. Obviously, this nonzero excitonic *σ* supports a TE mode surface wave as described in Eq. 1. Hence, this kind of electromagnetic wave is a typical propagating polariton mode.

We also deduced the Δ*k* dispersion for the atomically guided polariton mode from the measured Δ*k* dispersion. Specifically, we firstly calculated the dispersion relation for the evanescent wave of TE_00_ mode inside the silicon nitride waveguide by finite-difference time-domain method (see Methods). We then reconstructed the *E* − *k*_*WSe*2_ dispersion by summing the calculated *k_SiN_* and Δ*k*. For comparison, we theoretically analyzed the loss function of exciton–polariton by mode coupling theory (see Methods). In Fig. 2E, the obtained data are superimposed on the modeled loss function map. The anti-crossed dispersion apparently embodies a strong coupling between the 2 constituents (photonic mode and WSe_2_ B exciton) that hybridizes their dispersion relations. The best fitted g factor and Rabi splitting are 20 and 38 meV, respectively, and the photonic mode inside the monolayer waveguide is with a nearly constant effective mode index of about 2.6.

We next briefly discuss the potential application of the integrated interferometer. High-performance 2D-based integrated photonic devices, such as modulators [24–27], light emitters [28,29], and lasers [30–32], have been substantially investigated. By taking the 2D material itself as a waveguide, the interferometer provides new functions with compact footprints. In particular, compared to a conventional interferometer with horizontally configured beams (such as Mach–Zender interferometer), the vertical integration effectively shrinks device area [33]. It is worth of mentioning that we mainly operated the device in the visible light range, because most of the excitons of 2D materials are in this range. The waveguide and imaging facility are thus adapted to study the light coupling mechanism. Practically, devices in telecommunication bands (e.g., 1,550 nm) are of great interest. Far from the exciton energy, the results would be trivial, namely, the interference pattern is dispersionless and lossless. Future work on exploring real application devices may be carried in the infrared range but beyond the scope of this paper.

**Fig. 4.**
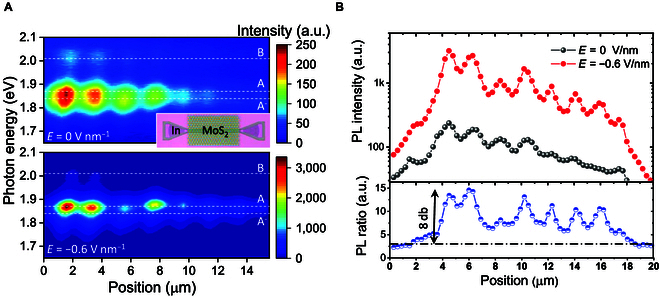
Demonstration of interferometric light modulation. (A) Selected spectral imaging of a MoS_2_-based vertically integrated waveguide (schematically shown in inset) at different gating. Corresponding gate electric fields are marked. A exciton, trion and B excion are marked by dash lines. (B) Upper panel: PL intensity along the waveguide. Bottom panel: PL intensity ration between the 2 PL profiles. The dash dot line illustrates the A exciton emission enhancement over the trion. Electromagnetic field modulation (8 dB) is obtained from the interferometer.

We use waveguide integrated modulator as an example to demonstrate controlling the atomically guided wave and the interference. Considering the common metal-oxide semiconductor used, the modulation speed may approach gigahertz range, similar as previously reported TMDC integrated waveguides (e.g., Ref. [9], MoTe_2_/graphene junction ~24 GHz; Ref. [27], WS_2_ ~0.3 GHz). Presently, we use a global imaging technique to study the coupling between monolayer waveguide and other photonic environment. It is challenging to dynamically characterize the operation bandwidth by our imaging setup. The response speed can be examined by employing local filed probe in future works. As illustrated in Fig. 4A (inset), the device is composed of a MoS_2_-based interferometer. A back gate voltage changes the carrier density of the TMDC channel. The gating results in double-fold effects. Firstly, the doping electrically switches the intrinsic exciton. A negative voltage turns the PL spectrum of MoS_2_ from trion dominated to exciton dominated (Fig. 4A) [34]. Secondly, we found that the applied electric field also redistributes the electromagnetic field inside the interferometer. Figure 4B summarizes the PL profile along the interferometer under different biases. The total intensity remarkably increases when approaching charge neutral point (MoS_2_ is naturally n-type doped). After deducting the quantum yield difference between trion and exciton, we still obtained a maximum electromagnetic field enhancement of about 8 dB inside the interferometer. We attribute this field modulation to a tunable evanescent coupling between the 2 waveguides, namely, the vdW gap as well as the evanescent filed inside are squeezed with increasing doping.

**Fig. 5.**
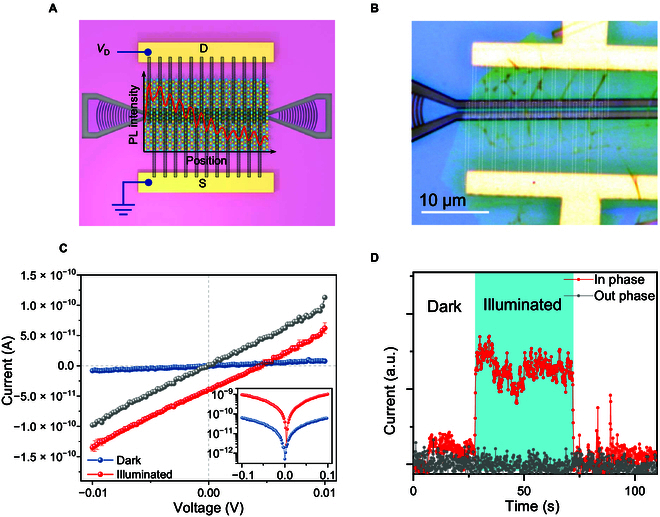
Demonstration of spectrally resolved photodetection. (A) Schematic of integrated interferometric photodetector. The dashed lines mark the boundaries of graphene contact. PL intensity under 514-nm laser excitation is superimposed on the MoS_2_ to indicate that the contact locates in-phase with the mode beating. (B) Optical micrograph of a device. Scale bar: 10 μm. (C) *I-V* curves of the detector at dark (blue curve) and 514-nm-light-illuminated (red curve) conditions. Photocurrent of a control test under 488-nm excitation (out phase) is also shown (gray curve). Inset: the *I-V* curves in log scale. (D) Zero-bias current at dark and illuminated condition, showing a unique photovoltaic-like wavelength-selective response can be obtained from surface wave interference.

We finally discuss spectrally resolved photodetection. Previously, 2D photodetectors integrated in a waveguide presents importantly enhanced photoresponse [35] due to the much longer absorption path along the in-plane direction [36–38]. We demonstrate a prototype interferometric photodetector that is able to resolve wavelength. Figure 5A illustrates the device, which consists of a vertically integrated interferometer with graphene interdigital electrodes fabricated by electron beam lithography (EBL) patterning, subsequently reactive ion etching. The 2 sets of electrodes were purposely placed on the peak and valley of the interference pattern at 514-nm excitation (or equally, in phase with the mode beating). To minimize extrinsic optical interference, we use monolayer graphene to form the contacts as demonstrated in Fig. 5B. For comparison, we also tested the device with a 488-nm laser, where the location of the electrodes are no longer aligned with the interference pattern (out phase with the mode beating). Generally speaking, photocurrent generates on TMDC phototransistor through photothermal or photogating effect. With symmetric contact, both of them require externally applying a bias voltage between source and drain electrodes [8], as shown in the out phase case (Fig. 5C).

In contrast, we observed a zero- bias photocurrent in the in phase case (Fig. 5D). We ascribed this photovoltaic like response to an asymmetric photothermal effect at the 2 contacts. That is, the electrode located on the oscillation peak generates larger photocurrent than the opposite one on the valley, resulting in a net photocurrent. Compared to its biased counterpart, this zero-bias photodetector present a much lower dark-noise figure. Because the noise source turns from shot noise to flicker noise when applying a bias voltage [39], obviously, this zero-bias photocurrent is wavelength sensitive. By fabricating a set of electrode samplers with variable spacing, an on-chip spectrometer similar to a stationary wave integrated Fourier transform spectrometry system could be achieved [40,41].

## Conclusion

In summary, we have achieved a vertically integrated interferometer with stacked 2D and silicon nitride waveguides. It distinctively reveals the optical coupling of atomically guided wave to other photonic modes. The new understanding highlight the crucial role played by unique evanescent coupling of 2D waveguide. Although the optoelectronic functionalities showcased in this work are still at early stage, the 2 simple instances pave a new avenue toward ultracompact and green chips. Moreover, our work inspires new strategy for multilayer photonic architecture and cross-layer coupling beyond traditional planar structure for fully monolithic 3D integrated photonics.

## Methods

### Device fabrication

The fabrication of the proposed TMDC/ Si_3_N_4_ waveguide was started from a 300-nm-thick Si_3_N_4_ slab grown on a 3-μm-thick SiO_2_ buried layer on a Si substrate. The Si_3_N_4_ waveguides and grating couplers (~8-dB coupling loss) were defined by EBL. Following inductively coupled plasma dry etching of Si_3_N_4_, the residual e-beam resist was removed using the piranha solution. The mono- or few-layer TMDC flakes were prepared using the mechanical exfoliation method from monocrystalline bulks, which were then transferred on top of the Si_3_N_4_ waveguides using a dry transfer method. For detector or modulator, 5-nm Cr/35-nm Au contacts were patterned with standard EBL, electron-beam evaporation, and lift-off processes. For spectral resolved photodetector, the interdigital graphene electrodes were fabricated by graphene transfer and EBL patterning, followed by an oxygen plasma etching process.

### Optical and electrical measurements

Real space PL images were obtained by a spectrometer system (ANDOR Shamrock 500i) equipped with a charge-coupled detector (iDus DU420A-BEX2-DD, cooled down to −70 °C). A supercontinuous laser (OYSL, SC-Pro-7) with acousto-optic tunable filter (OYSL, AOTF0052) was used as excitation source (at a power level ~1 mW). Note that for the measurements with excitation energy very near B exciton, 2 oscillation periods are observed. We ascribe them to the upper and lower branches of the polariton. They mix due to the filter of the supercontinuum laser is of relatively broad width. Electrical characteristics were measured by a semiconductor analyzer (Primarius, FS Pro). All measurements were performed at room temperature under ambient conditions.

### Finite-difference time-domain method

We analyze the photonic mode and the effective refractive index of the hybrid waveguide using a finite-element-method-based simulation by employing the commercial finite-element package COMSOL Multiphysics. In the hybrid system, a MoS_2_ monolayer is deposited on a Si3N4 waveguide (width: 500 nm, height: 300 nm). The Si_3_N_4_ waveguide is supported by a SiO_2_ substrate. The excitation wavelength is set to 400 to 700 nm in the simulations. The refractive index of Si_3_N_4_ is 2. We employ electromagnetic waves module and the eigenvalue solver to calculate the waveguide photon modes. The eigenvalues are obtained, whose real and imaginary parts show the effective refractive index and propagation distance, respectively. We also calculate the electric field distribution in the cross-section of the hybrid waveguide.

### Loss function of exciton–polariton by mode coupling theory

We employ a classical model of coupled oscillators to describe the coupling of waveguide photons (Eq. 3) and the TMDC excitons (Eq. 4) [42–44]. We assume that only the waveguide photon is driven.

$$\ddot{x_{1}}+\gamma_{1}\dot{x_{1}}+\omega_{1}^{2}x_{1}+2g\dot{x_{2}}=F$$ (3)

$$\ddot{x_{2}}+\gamma_{2}\dot{x_{2}}+\omega_{2}^{2}x_{2}+2g\dot{x_{1}}=0$$ (4)

The solution of the equations has a plane wave form

$$x_{1}=\frac{f∙\Gamma_{2}}{\Gamma_{1}\Gamma_{2}+K^{2}}e^{-\mathrm{i\omega t}},x_{2}=\frac{f∙K}{\Gamma_{1}\Gamma_{2}+K^{2}}e^{-\mathrm{i\omega t}}$$ (5)

where *f ∝ F, Γ_j_ = ω_j_^2^−iω_j_−ω^2^, K = 2igω*. The polarization induced by the hybridization of different modes therefore is proportional to *x*_1_ and thus the dispersion map can be visualized as Im(*x*_1_).

## Data Availability

All data needed to evaluate the conclusions in the paper are present in the paper and/or the Supplementary Materials. Additional data related to this paper may be requested from the authors.
