# Supplementary material for: Optical Coupling in Atomic Waveguide for Vertically Integrated Photonics
Source: Research (Wash D C). 2024 Mar 11;7:0329. doi: 10.34133/research.0329 (PMC10927546; doi:10.34133/research.0329)
Supplement: Supplementary 1 — Supplementary Text 1 to Supplementary Text 3 Figs. S1 to S4 [file research.0329.f1.docx]

Supplementary Materials for

**Optical Coupling in Atomic Waveguide for Vertically Integrated Photonics**

Yue Wang^1†^, Junzhuan Wang^1†^, Ruijuan Tian^2^, Jiapeng Zheng^3^,

Lei Shao ^4*^, Bo Liu^5*^, Fengqiu Wang^1^, Xuetao Gan^2*^,

Yi Shi^1^ and Xiaomu Wang^1*^

^1^ School of Electronic Science and Engineering, Nanjing University, Nanjing 210093, China

^2^ Key Laboratory of Light Field Manipulation and Information Acquisition, Ministry of Industry and Information Technology, and Shaanxi Key Laboratory of Optical Information Technology, School of Physical Science and Technology, Northwestern Polytechnical University, Xi’an 710129, China.

^3^ Department of Physics, The Chinese University of Hong Kong, Hong Kong SAR, China

^4^ State Key Laboratory of Optoelectronic Materials and Technologies, Guangdong Province Key Laboratory of Display Material and Technology, School of Electronics and Information Technology, Sun Yat-sen University, Guangzhou 510275, China

^5^ Institute of Optics and Electronics, Nanjing University of Information Science and Technology, Nanjing 210044，China

† These authors contributed equally to this work

*Corresponding authors: xiaomu.wang@nju.edu.cn, xuetaogan@nwpu.edu.cn, bo@nuist.edu.cn, shaolei5@mail.sysu.edu.cn

Supplementary Text 1

MoS_2_ and Graphene based vertically integrated interferometer

Experimentally, we have prepared and tested several different types of 2D materials such as other TMDC (MoS_2_ and WS_2_ et al) and graphene. And similar results were observed, we have presented WSe_2_ in the maintext. The results of WSe_2_ is more straightforward because there are two prominent excitons (A exciton at 1.65eV and B exciton at 2eV). We used PL emission of WSe_2_ A exciton as a probe to detect the spatial light field distribution of B exciton-polariton with higher energy.

For other TMDC such as MoS_2_, there is only one prominent exciton (and the weak B exciton is also too near to the A exciton). Low energy polariton (wavelength longer than 660nm) can not be probed by PL, hence it is hard to directly mapping the interference. We accordingly fabricated a MoS_2_/hBN/WSe_2_ heterostructure in which MoS_2_ is close to the SiN waveguide and the WSe_2_ probe is separated ~10nm away from the interferometer by the hBN spacer. The hBN ensures WSe_2_ only acts as a probe to detect the interference but do not participate it. **Fig. S1** shows the main result. It is nearly the same as the WSe_2_ case, except the polariton crossover energy is different (~1.87eV at MoS_2_ A exciton).

While the physics of TMDC is quite similar, graphene differs from TMDC due to the different optical properties. We also fabricated and tested a graphene based vertically integrated interferometer. The device is with similar device as shown in the maintext, except here a monolayer graphene is used as 2D waveguide. **Fig. S2A** shows the device photo, where the graphene used is shown in **Fig. S2B**. Different from TMDC waveguide, we used Raman 2D peak to probe the interference pattern. **Fig.S2C** shows the Raman mapping of the device and **Fig. S2D** summarizes the profile along monolayer graphene. Clear period oscillation is also observed in the monolayer graphene/silicon nitride waveguide stacking. For graphene, a Drude weight intrinsically provides an optical conductivity σ in equation (1). And the smaller σ results in lower loss (flatten valley base line as shown in **Fig. S2D**) and different interference period compared to the WSe_2_ case shown in the maintext (**Fig. S2E**).

We found the interference behavior is universal among different 2D materials. And the interference pattern depends on the optical band and optical properties of specific materials.

Supplementary Text 2

Evanescent coupling of the vertically integrated interferometer

The interferometer can be only observed in thin samples. We tested multi-layer devices where MoS_2_ are assembled layer by layer as shown in **Fig. S3 A&B**. Thicker samples present faster decay. In addition, the magnitude of the interference pattern rapidly decreases. For sample layer number larger than 5, the oscillation almost disappear (**Fig. S3C**). The results clearly highlight the crucial role played by surface wave in the interferometer. That is, with increasing thickness, the multilayer MoS_2_ confine more light inside the material and turns to significantly lossy. Consequently the interference no longer happens as evanescent waves vanish.

Supplementary Text 3

Polarization effect for light coupling of the vertically integrated interferometer

We also investigated the TE and TM polarization coupling of the vertically integrated interferometer. **Fig.S4A** shows the photo of the device with WSe_2_ film on the waveguide and illustration of the TE and TM polarized light scheme using a 514 nm laser. For our system, TE00 is the ground mode, i.e. the mode with the highest coupling efficiency. In fact, the TM00 mode has similar results to TE, but with lower efficiency due to the different boundary conditions between the electric and magnetic fields. The results of WSe2 excited by TE and TM polarization light are presented in **Fig.S4 B&C**. The periodic patterns observed are similar for both polarizations, and they are attributed to mode-beating between 2D and (different) silicon nitride waveguide modes. However, the periods differ slightly, with 1.99 μm for TE and 1.63 μm for TM. This difference is due to the slightly different effective index for TE and TM modes.


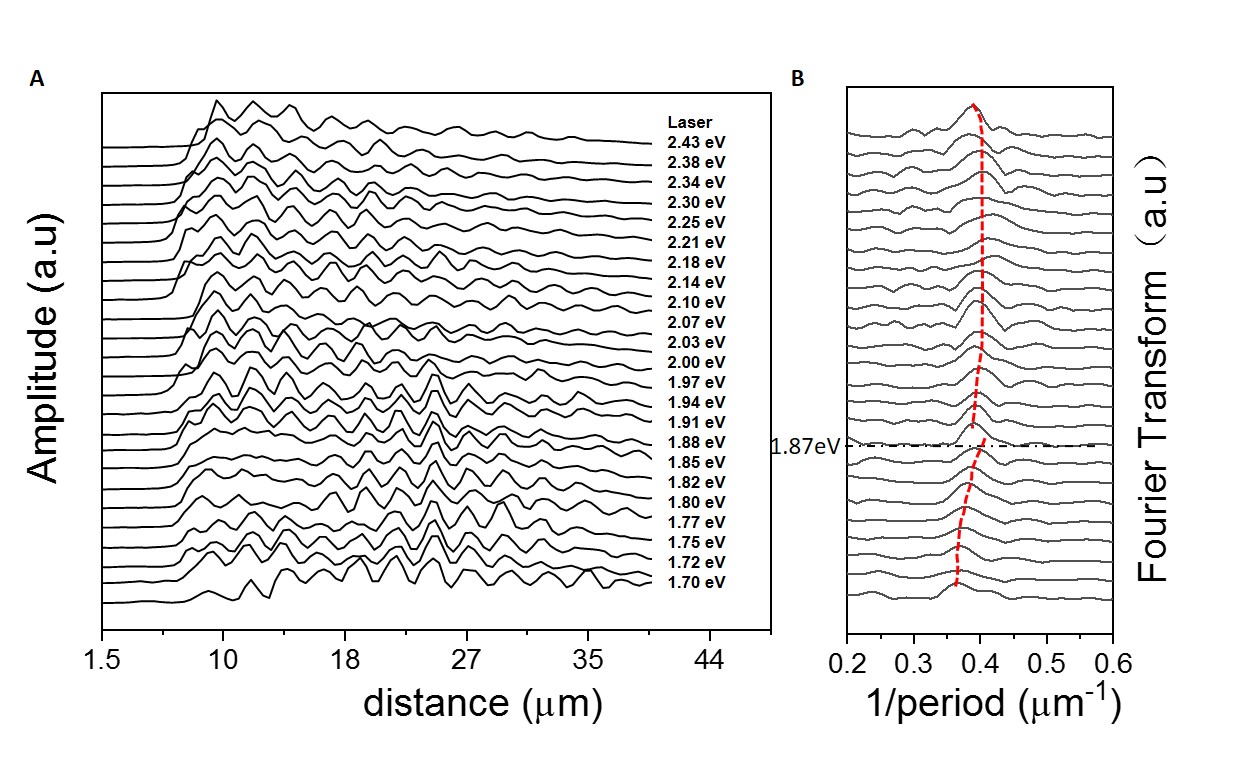


**Fig.S1 Interference of MoS_2_ polariton. (A)** Real space PL profiles of a typical MoS_2_ vertically integrated interferometer taken at various excitation wavelength (490-730nm). All the profiles are displaced vertically for clarity. **(B)** The corresponding Fourier transform profiles. The dashed lines guide the main peaks. And the dot-line indicates the exciton energy of MoS_2_. The anti-crossover behavior signals the formation of a polariton.


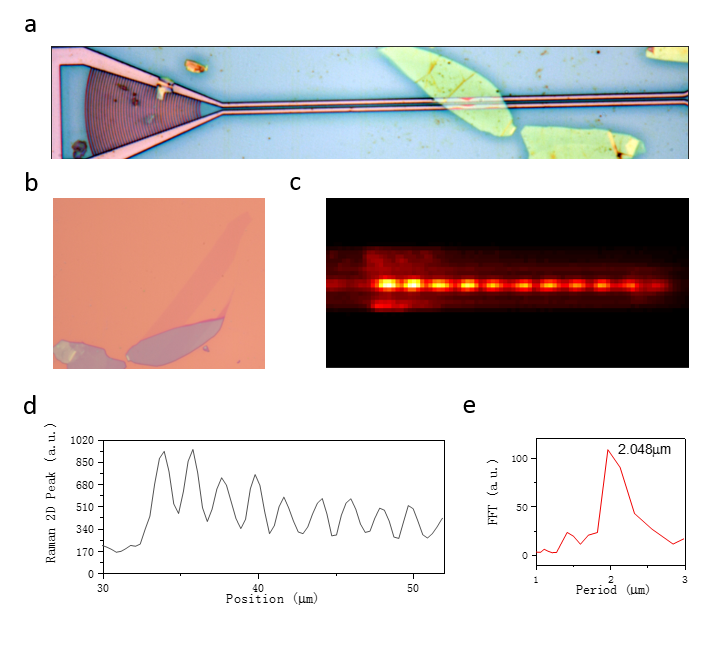


C

E

D

B

A

**Fig. S2. Graphene based vertically integrated interferometer**. **(A)** Micro photograph of a typical device. **(B)** The monolayer graphene sample used in (A). **(C)** Real-space Raman 2D peak imaging of the device shown in (A), excited by 514nm Argon laser. **(D-E).** Real-space Raman profiles (D) and the corresponding Fourier transform profiles (E) of the graphene based vertically integrated interferometer.


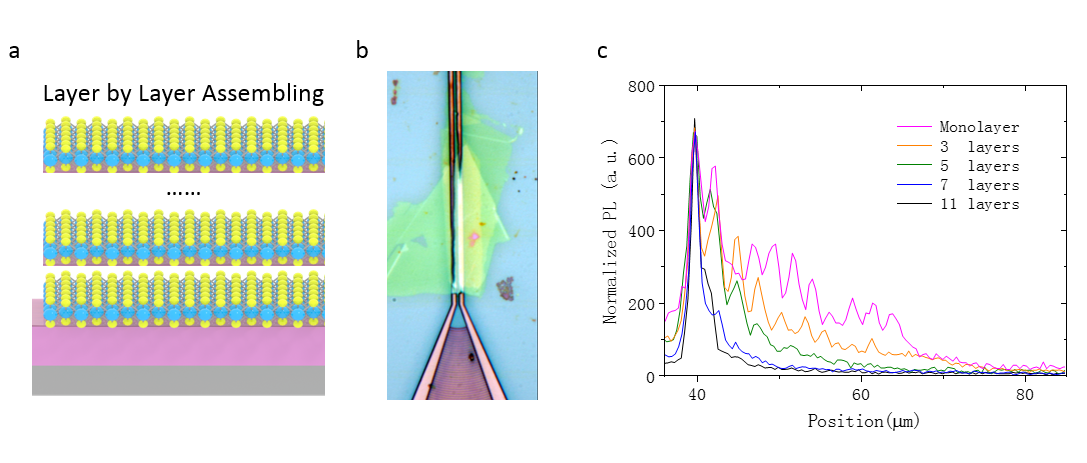


C

B

A

**Fig. S3. Evanescent coupling of the vertically integrated interferometer.** (A-B), Schematics (A) and micro photograph (B) of a typical layer-by-layer assembled multilayer device. **(C)** Real-space PL imaging of the device shown in (B), excited by 514 nm Argon laser. The interference vanishes with increasing layer number.


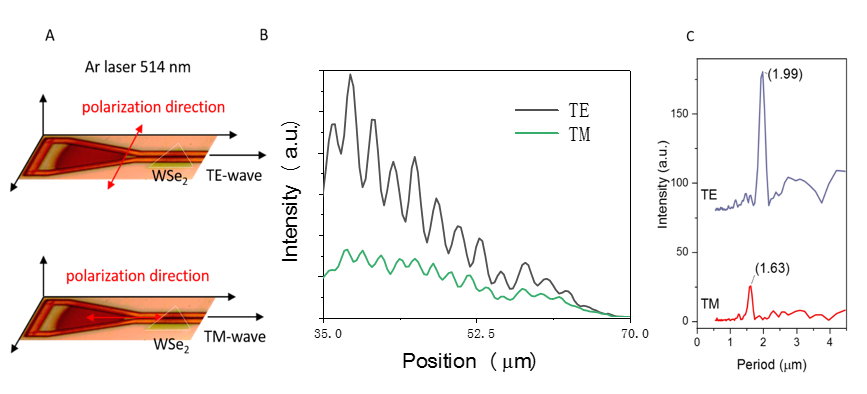


**Fig.S4 Interference of MoS_2_ polariton.** (A) Photo of the device with WSe2 film on the waveguide and illustration of the TE and TM polarised light scheme using a 514 nm laser. B-C. TE and TM mode PL of the WSe_2_ film along the waveguide (B) and corresponding period (C).
